# Supplementary material for: The interactome of intact mitochondria by cross-linking mass spectrometry provides evidence for coexisting respiratory supercomplexes
Source: Mol Cell Proteomics. 2017 Dec 8;17(2):216–32. doi: 10.1074/mcp.RA117.000470 (PMC5795388; doi:10.1074/mcp.RA117.000470)
Supplement: Supplemental Data [file supp_RA117.000470_133922_0_supp_23978_fzffwf.zip › spectra_annotation/mito_spectra_annotation/191-1-1-1-7-1.pdf]

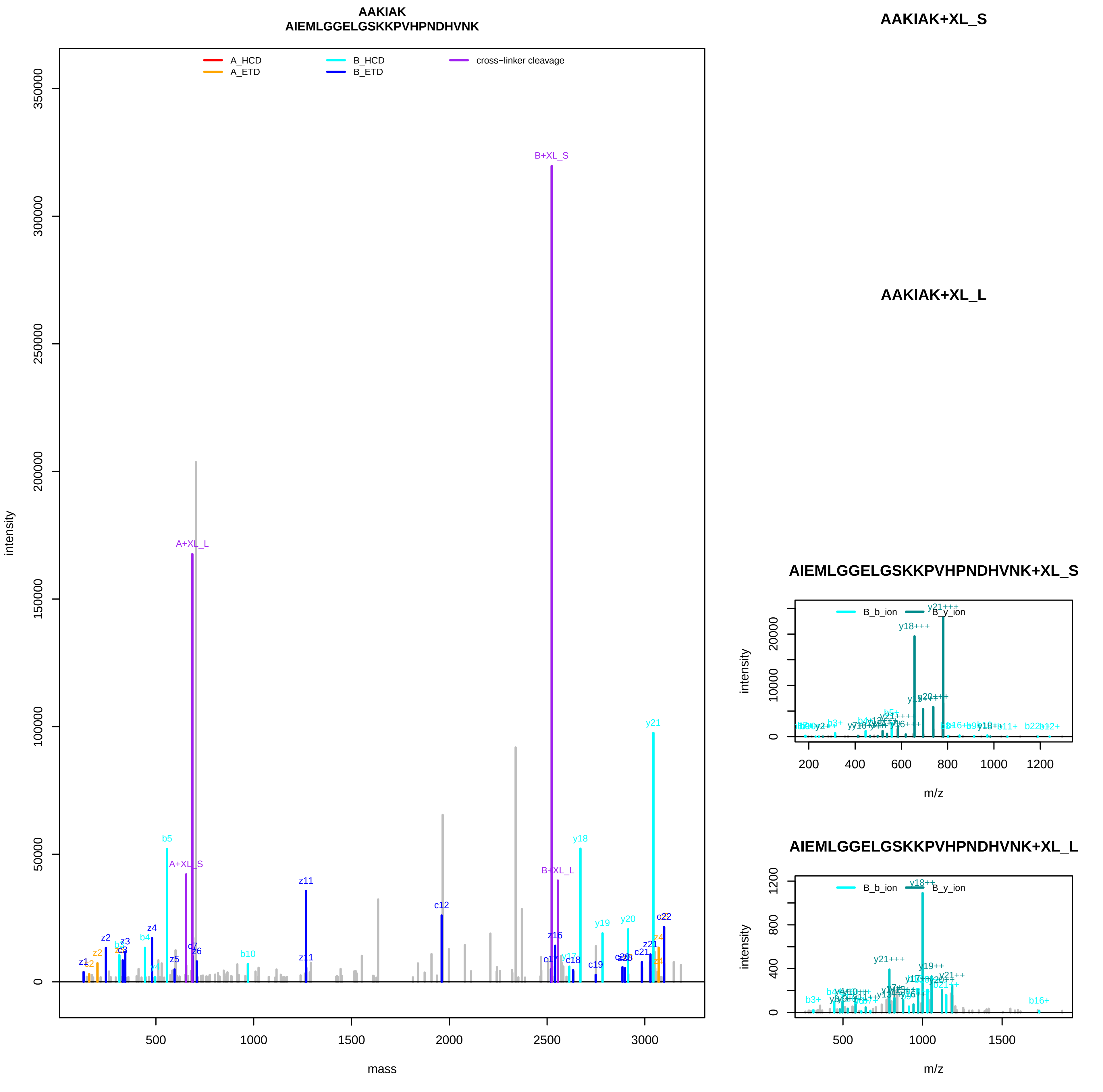
AAKIAK
AIEMLGGELGSKKPVHPNDHVNK
A_HCD
A_ETD
B_HCD
B_ETD
cross−linker cleavage
intensity
mass
0
50000
100000
150000
200000
250000
300000
350000
500
1000
1500
2000
2500
3000
B+XL_S
A+XL_L
A+XL_S
B+XL_L
b5
y18
y21
y20
y19
z11
c12
c22
z16
z4
z2
z3
z1
b4
b10
c7
z6
z5
c18
c19
c21
z21
AAKIAK+XL_S
AAKIAK+XL_L
AIEMLGGELGSKKPVHPNDHVNK+XL_S
B_b_ion
B_y_ion
y21+++
y18+++
y20+++
m/z
0
10000
20000
200
400
600
800
1000
1200
AIEMLGGELGSKKPVHPNDHVNK+XL_L
B_b_ion
B_y_ion
y18++
y21+++
y19++
b16+
b3+
0
400
800
1200
500
1000
1500
m/z
